# Novel Treatment of Disseminated Coccidioidomycosis in a Dog with Voriconazole

**DOI:** 10.1155/2018/1785748

**Published:** 2018-04-05

**Authors:** Chantel Raghu, Christopher Reagh

**Affiliations:** ^1^University of Wisconsin Veterinary Care, 2015 Linden Drive, Madison, WI 53706, USA; ^2^Veterinary Emergency Service, 1612 N High Point Rd, No. 100, Middleton, WI 53562, USA

## Abstract

A dog with disseminated coccidioidomycosis involving the vertebral, cutaneous, and pulmonary systems was treated successfully with voriconazole after failing traditional therapy with fluconazole and terbinafine. This report is the first to describe the successful management of refractory coccidioidomycosis with voriconazole in a dog.

## 1. Introduction

*Coccidioides* spp. is a dimorphic soilborne fungus endemic in the southwestern United States and parts of Mexico and Central and South America that can infect both humans and animals [1]. The organism can remain dormant within its host for several years after travelling to an endemic area, and there have been rare reports of coccidioidomycosis in humans in Europe [2–7]. Coccidioidomycosis can be difficult to diagnose if a thorough travel history is not investigated. Clinical signs in dogs include persistent or fluctuating fever, anorexia, weight loss, lameness, draining skin lesions, hyperesthesia, uveitis, and acute blindness [1]. Dogs with disseminated coccidioidomycosis carry a grave prognosis despite standard treatment with fluconazole with or without terbinafine. This case report is the first to describe successful management of disseminated coccidioidomycosis in a dog with the novel triazole compound voriconazole.

## 2. Case Description

A 9-year-old, 18.1 kg, spayed female, Blue Heeler-Cross dog was presented to the University of Wisconsin Veterinary Care (UWVC) for a draining cutaneous lesion on the right flank, intermittent fever, spinal pain, pelvic limb paresis, and muscle wasting, progressive over 12 months. A year prior to presentation the draining cutaneous tract was biopsied and cultured by the primary care veterinarian. Histopathology showed pyogranulomatous inflammation with severe vasculitis, and aerobic and anaerobic bacterial cultures were negative. Thoracic radiographs showed normal pulmonary parenchyma with no evidence of fungal granulomas. Urine was submitted for* Blastomyces* quantitative sandwich enzyme immunoassay (EIA; Miravista Diagnostics) and was negative. Pentoxifylline [22 mg/kg orally every 8 hr (Mylan Pharmaceuticals Inc., Morgantown, West Virginia, USA)] had been administered for 30 days to treat vasculitis, with minimal improvement in the draining tract. Anti-inflammatory prednisone [1 mg/kg orally every 24 hr (Roxane Laboratories Inc., Columbus, Ohio, USA)] resulted in substantial improvement in the fever and lesion drainage. As the prednisone dosage was tapered to 0.25 mg/kg orally every 48 hours, the fever returned and the draining tract worsened. Travel history included west Texas and northern California, but the dog had resided in Iowa and Wisconsin for the preceding four years.

On physical examination at UWVC, the dog was quiet, alert, responsive, and hydrated, with pink mucous membrane, a normal capillary refill time, and normal heart and respiratory rates. The rectal temperature was elevated at 104.1°F. The dog was paraparetic and was in pain upon thoracolumbar spinal palpation. An alopecic lesion with thickened dermis and a draining tract producing serosanguinous and mucopurulent fluid was present in the right flank. A fluctuant subcutaneous mass was palpable between ribs 12 and 13 in the left hypaxial area. There was no evidence of retinal abnormalities, mucocutaneous lesions, or long bone pain, and the dog was neurologically appropriate. The remainder of the physical examination was within normal limits.

Given the history and clinical presentation, a primary differential diagnosis for the thoracolumbar pain, fever, draining tract, and fluctuant subcutaneous mass was a migrating foreign body with possible secondary bacterial infection. Cytological examination of fluid from the left hypaxial fluctuant mass showed mixed inflammation, with nondegenerate neutrophils predominating and no microorganisms seen. An aerobic bacterial culture of fluid from the draining tract grew* Staphylococcus pseudintermedius* and* Streptococcus dysgalactiae*. A complete blood (cell) count (CBC) revealed mild normocytic normochromic anemia (hematocrit: 0.35 L/L; reference interval: 0.39 to 0.57 L/L), with a mild leukocytosis consisting of mature neutrophilia (neutrophils: 12.2 × 10^9^/L, reference interval: 2.6 to 10.0 × 10^9^/L). The serum biochemical panel was normal except for elevated globulins (49 g/L; reference interval: 22 to 35 g/L), consistent with an inflammatory response.

A CT scan of the chest and abdomen was performed under general anesthesia and revealed pulmonary nodules with mildly enlarged sternal and cranial mediastinal lymph nodes. There was mild permeative to moth-eaten lysis of the cranioventral aspect of the vertebral body of T13, consistent with osteomyelitis. Multiple fluid pockets were present in the subcutaneous tissues, with the largest within the left hypaxial muscles at the level of T13 and in the subcutaneous tissues immediately lateral to the right pelvic inlet.

The dog was treated with amoxicillin/clavulanic acid [25 mg/kg PO q8 hr (Augmentin; GlaxoSmithKline, Philadelphia, Pennsylvania, USA)] to treat a bacterial infection suspected to be secondary to a migrating cutaneous foreign body that could no longer be detected. The owners were instructed to treat the dog for a total of 2 months and to monitor lameness, body temperature, and draining tracts.

The dog continued to have febrile episodes, trembling, and cutaneous drainage despite treatment with amoxicillin/clavulanic acid. The dog also developed right hind limb lameness and a new soft tissue swelling at the level of the right tarsus. The dog represented to UWVC for reevaluation 6 months after initial presentation. Physical exam findings were similar to initial presentation with the addition of a right tarsal swelling. A repeat CT scan of the chest and abdomen showed static pulmonary nodules and the development of pleural effusion. The vertebral bodies of T13 and L1 had progressive lysis consistent with progressive osteomyelitis. The previously noted fluid pocket within the left hypaxial muscle at the level of T13 had enlarged in size, but the caudal right fluid pockets had decreased in size.

An undetected persistent foreign body was suspected, and the left caudal thorax and abdomen were explored surgically. An elliptical incision was made around the fluctuant subcutaneous swelling over the cranial left flank near the 13th rib, and the surrounding subcutaneous tissue was dissected to isolate the abnormal tissue, which was excised and submitted for histopathology. Below the swollen tissue, multiple draining tracts were identified and followed communication with the thoracic cavity was documented, but no foreign body could be identified. The peritoneum, abdominal musculature, and subcutaneous tissue were closed and a Jackson-Pratt drain was placed at in the cranial left flank. The skin was closed using staples.

**Figure 1:**
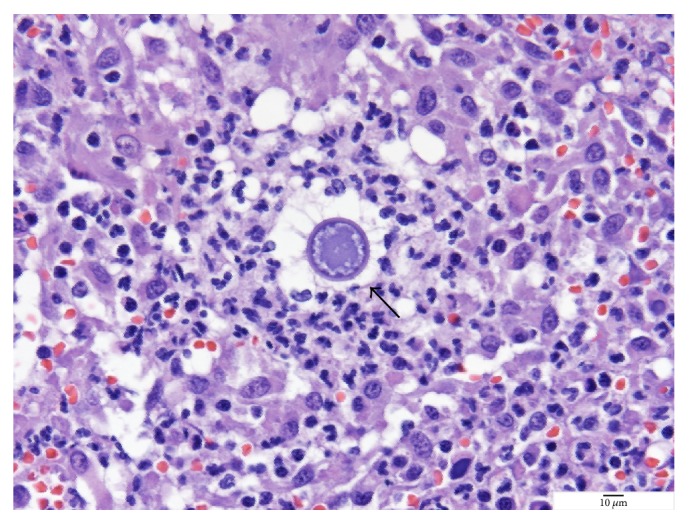
Immature spherule at the late segmentation phase prior to development of endospores of* Coccidioides immitis* (arrow) isolated from the left flank mass. The round structures are approximately 30–40 um in diameter with 2 um thick pale basophilic cell walls and heterogeneous amorphous pale amphophilic central material. Periodic acid Schiff (PAS) stain.

Histopathology of the abnormal tissue showed marked locally extensive pyogranulomatous and lymphoplasmacytic dermatitis and cellulitis with draining tracts and intralesional fungal conidia. The fungal conidia were round structures approximately 30–40 micrometers in diameter with 2 micrometers of thick pale basophilic cell walls and heterogeneous amorphous pale amphophilic central material, consistent with immature* Coccidioides immitis* spherules (Figure 1). These findings were consistent with a diagnosis of disseminated coccidioidomycosis with cutaneous, vertebral, and pulmonary involvement. To enable future clinical monitoring, serum was submitted for detection of* Coccidioides* antibodies by agar gel immunodiffusion (IDEXX Laboratories), and the serum antibody titer was 1 : 32.

Treatment with fluconazole (Harris, Fort Myers, Florida, USA), 7.7 mg/kg PO, and q12 h was started along with terbinafine (Camber, Piscataway, New Jersey, USA), 27 mg/kg, PO, and q24 h. Anecdotally, terbinafine has been proposed to have synergistic effects when added to fluconazole in the treatment of* Coccidioides* [8]. Over the next 4 weeks, the previous dosage of prednisone was tapered and discontinued.

The patient's response to antifungal therapy was evaluated at the initiation of and throughout treatment using a modified Mycosis Study Group (MSG) score that is used in human patients (Table 1). The original MSG score takes into account clinical signs, radiographic imaging, and antibody titer and generates a composite score [9–11]. A modified MSG score that omits the radiographic component was used in this dog as has been described previously [9, 10], since the dog's lesions were best documented using a CT scan and repeated CT scans under anesthesia to monitor treatment response were not considered in the dog's best interest. Thoracic radiographs were considered to be an inaccurate substitution for a CT scan in this case, since the pulmonary nodules seen on CT were below the limit of detection on the baseline radiographs performed by the primary care veterinarian.

During treatment with fluconazole and terbinafine, the dog developed inappetence, which resolved when terbinafine was discontinued after 4 weeks of treatment. After 3 months of fluconazole treatment, the dog's energy level had improved and the cutaneous lesion was no longer draining, although there was a fluctuant subcutaneous swelling at the site of the previous tract. The dog continued to have febrile episodes with trembling, but they were less frequent. The* Coccidioides* antibody test showed a rising titer (1 : 64) despite fluconazole treatment. Recheck chemistry panel was normal except for persistently elevated globulins (50 g/L; reference interval: 22 to 35 g/L) and mildly elevated serum alkaline phosphatase (270 U/L; reference interval: 20 to 157 U/L). As a result of the persistent fever, rising antibody titer, and a MSG score that was classified as unresponsive, rescue therapy with liposomal amphotericin B infusions and voriconazole was offered to the owner. The owner declined hospitalization for amphotericin B but elected to start voriconazole (Glenmark, Mahwah, New Jersey, USA), at 2.7 mg/kg, PO, q12 h, on an empty stomach. One week after starting voriconazole, a serum trough concentration was measured at 1.7 mcg/mL, which is within the therapeutic range targeted in humans with systemic mycoses (reference interval: 1.0–6.0 mcg/mL; the University of Wisconsin Health and Clinics Clinical Laboratories in Madison, Wisconsin) [12, 13].

After 3 months of voriconazole treatment, the dog was consistently afebrile with no draining tracts, resolved paraparesis, and a normal energy level and appetite. The remaining clinical signs were pain upon thoracolumbar spinal palpation and a fluctuant swelling on the right flank cranial to the location of the draining tract. Repeat* Coccidioides* antibody titer at 6 months after diagnosis and 3 months after starting voriconazole was at 1 : 32. Chest radiographs showed normal pulmonary parenchyma with no nodules and the absence of pleural effusion. Recheck voriconazole serum trough concentration was submitted to the Fungus Testing Laboratory at the University of Texas Health Science Center in San Antonio, Texas, and was considered therapeutic at 2.01 mcg/mL (reference interval: 1.0 to 6.0 mcg/mL). A serum biochemical panel showed new hypoalbuminemia (16 g/L; reference interval: 27 to 39 g/L) and continued but stable hyperglobulinemia (46 g/L; reference interval: 24 to 40 g/L). Alkaline phosphatase had improved (191 U/L; reference interval: 5 to 160 U/L) from the previous biochemical panel. Because of the severity of the hypoalbuminemia, additional testing was performed; serum bile acids were within normal limits, (1.6 *μ*mol/L preprandial; reference interval: 0 to 6.9 *μ*mol/L; 6.2 *μ*mol/L postprandial; reference interval: 0 to 14.9 *μ*mol/L), urine was negative for proteinuria, and a baseline cortisol was only modestly decreased at 38.6 nmol/L (reference interval: 55.1 to 165.54 nmol/L). An ACTH stimulation test was not performed because the negative predictive value for hypoadrenocorticism remains high at the cut-point of a baseline cortisol ≥40 nmol/L, [14] and azoles are known to suppress endogenous cortisol concentrations [15]. The dog was not clinically treated for hypoalbuminemia and FAST scan of the thorax and abdomen was negative for free fluid. Overall, the patient's modified MSG score was improved based on the amelioration of clinical signs and decreased* Coccidioides* antibody titer.

After 6 months of voriconazole treatment, the fluctuant right flank swelling was enlarged and was turgid upon palpation. The voriconazole serum concentration was below therapeutic range (0.60 mcg/mL; reference interval: 1.0 to 6.0 mcg/mL). The voriconazole dosage was increased from 2.7 mg/kg to 4.1 mg/kg twice daily. Recheck serum voriconazole concentrations after one week of this higher dose were within the therapeutic range (1.25 mcg/mL; reference interval: 1.0 to 6.0 mcg/mL). Repeat* Coccidioides* antibody titer was stable at 1 : 32. Serum biochemical panel showed static to mildly improved albumin (19 g/L; reference interval: 27 to 39 g/L) and stable hyperglobulinemia (46 g/L; reference interval: 24 to 40 g/L). The ALP was mildly increased to twice the upper limit of the normal range (336 U/L; reference interval: 5 to 160 U/L), which was suggestive of cholestasis secondary to azole therapy. The bilirubin and ALT remained within normal limits. The patient's modified MSG score after increasing the voriconazole dose was stable based on continued resolution of the fever, resolved skin drainage, improved muscle mass, and stable* Coccidioides* antibody titer. At the time of writing, 13 months after diagnosis of disseminated coccidioidomycosis and 7 months into voriconazole therapy, the dog continues to feel well and remains free of fever or draining tracts, with normal energy and pelvic limb strength, with the exception of residual thoracolumbar pain and subcutaneous swelling in the right flank.

## 3. Discussion

Disseminated coccidioidomycosis was an unexpected diagnosis in this dog because of the lack of recent travel to endemic areas within the past four years.* Coccidioides* is endemic in some areas of the southwestern United States, Mexico, and South America, and it was most recently found in south central Washington [16]. The Center for Disease Control and Prevention (CDC) in Wisconsin was contacted to report the disease and evaluate the possibility of recent exposure to* Coccidioides* in the Midwest. The CDC confirmed that* Coccidioides* is not endemic in Wisconsin and that it is most likely that the dog inhaled infectious arthroconidia while living in west Texas. In humans,* Coccidioides* can remain dormant for several years and can become an active infection as the patient ages or becomes immunocompromised. This case report further highlights the importance of a detailed travel history beyond the past four years and consideration of infectious diseases that are nonendemic to the present residence.

The treatment of choice for* Coccidioides* in humans depends on severity, chronicity, and anatomic involvement. A mild respiratory syndrome may resolve on its own, whereas a chronic pulmonary or disseminated disease warrants antifungal therapy [17]. In humans, fluconazole is the agent of choice and amphotericin B is often used for rapidly progressive coccidiodal infections [17, 18]. Newly available antifungal drugs that may be beneficial in refractory cases include voriconazole, caspofungin, and posaconazole [10, 17].

In dogs, management of coccidioidomycosis involves long-term antifungal drug treatment, typically with azoles such as ketoconazole, itraconazole, or fluconazole [1]. Fluconazole is the most widely prescribed azole to treat coccidioidomycosis because it is well absorbed in the gastrointestinal tract even in anorexic animals, may be less hepatotoxic than other azoles, [19, 20] and is supplied in an affordable generic formulation [21]. Terbinafine has been anecdotally recommended for possible synergistic activity when combined with fluconazole, but this has not been evaluated clinically in dogs [1, 8]. Amphotericin B is recommended in cases of severe, diffuse pulmonary infections to achieve a faster onset of action when compared to azoles, or if azoles are not tolerated in the individual patient [1]. There have been a few case reports and one case study documenting the successful treatment of disseminated coccidioidomycosis in human patients [10, 22–24]. To the authors' knowledge, this is the first report of successful management of refractory disseminated coccidioidomycosis with voriconazole in a dog.

Voriconazole is a relatively new antifungal agent that is a derivative of fluconazole, having one triazole moiety replaced by a fluoropyrimidine ring and a methyl group added to the propanol backbone [25]. Its primary mechanism of action is inhibition of fungal cytochrome P450-dependent 14*α*-sterol demethylase, which is an essential enzyme in ergosterol biosynthesis [26]. A study on the disposition of voriconazole in healthy dogs showed good oral bioavailability (>75%) but that chronic oral dosing can lead to lower systemic concentrations in the dog [27], likely due to cytochrome P450 autoinduction of metabolism. Peak levels occur approximately 3 hours after oral dosing, and the volume of distribution is approximately 1.3 L/kg [27].

Voriconazole is currently labeled by the United States Food and Drug Administration (FDA) for invasive aspergillosis and salvage therapy for* Scedosporium* and* Fusarium* infections in humans. Voriconazole has previously been cost prohibitive in dogs, and there are few reports of its successful use in canines to treat* Byssochlamys* sp., intracranial* Cladophialophora* sp., and CNS aspergillosis, and topically for ocular* Malassezia pachydermatis*,* Scedosporium* sp.,* Aspergillus* sp., and* Candida* sp. [28–33]. In this dog with disseminated coccidioidomycosis, voriconazole was used at a dose of 2.7 mg/kg by mouth twice daily and was successful in improving clinical signs, stabilizing the antibody titer, and maintaining therapeutic serum concentrations for the first six months of therapy. After 6 months, the dose was increased to 4.1 mg/kg twice daily to maintain a therapeutic concentration. The starting dosage was lower than previously recommended based on anecdotal clinical experience with neurologic adverse effects (obtunded mentation and lethargy) in other patients treated with voriconazole at an initial dose of 4 mg/kg by mouth twice daily [34].

The dog in this report tolerated the voriconazole well with a normal appetite, no vomiting or diarrhea, improved body weight, and no neurologic adverse effects. Common adverse effects in humans include visual disturbances and dermatologic reactions such as photosensitivity [35]. The incidence of elevated liver values (ALT, AST, and ALP) was found to be 14.6% in a retrospective study in 46 humans who received intravenous or oral voriconazole to treat invasive fungal infections [36]. Although the incidence of elevated liver enzymes was relatively high, voriconazole was discontinued in only 3 out of the 46 patients due to hepatotoxicity [36]. The dog in this case report showed a mild subclinical increase in ALP activity after six months of voriconazole therapy. The reason for the development of hypoalbuminemia during voriconazole treatment in this dog is not clear. Voriconazole has not been known to cause hypoalbuminemia in humans, and preclinical toxicity studies in dogs and rodents administered voriconazole orally for up to two years and intravenously for up to six months did not demonstrate hypoalbuminemia [35, 37]. The severity of the hypoalbuminemia in this dog was more dramatic than one would expect from chronic inflammatory disease, but the concurrently elevated globulins could be supportive of this mechanism. Monitoring of both liver enzymes and serum albumin appears to be warranted in dogs treated with voriconazole going forward.

In summary, this case report demonstrates the successful use of voriconazole in a dog with refractory disseminated coccidioidomycosis to ameliorate clinical signs and improve the modified MSG score. Due to the severity of the patient's initial disseminated disease, it is not clear how long the dog will require antifungal treatment. In people, disseminated coccidioidomycosis involving bone is treated with an azole for a minimum from 3 years to lifelong [38]. Serology is a useful tool to monitor therapy and should decline with effective treatment [39]. A low (≤1 : 4) or undetectable antibody titer suggests control of fungal growth, but up to 30% of human patients will relapse after discontinuation of therapy [39, 40]. Since voriconazole can autoinduce its own metabolism in dogs, trough serum concentrations are recommended to monitor subtherapeutic or toxic levels, starting from one week after initiation and every three months thereafter. Voriconazole shows some promise as salvage therapy for coccidioidomycosis in dogs, but further clinical experience and study are warranted.

## Figures and Tables

**Table 1:** *Modified MSG score*. The MSG score is a composite score, comprising the sum of points assigned by the following characteristics: (1) a clinical signs score, with 1 point given for each sign listed; (2) a radiographic imaging scoring system, with points assigned for the size of the abnormality, the presence of satellite lesions, associated adenopathy, and changes in size or presence of infiltrates; and (3) serology points, with a complement fixation titer of 1 : 2 assigned 1 point, 1 : 4 assigned 2 points, 1 : 8 assigned 3 points, and so forth. The patient's response to treatment was classified as either improved, stable, or unresponsive. An improved outcome was defined as one of the following: (1) a score ≤50% of the pretreatment composite score; (2) the unequivocal documentation of clinical improvement; or (3) a 25–49% decrease in the MSG score and the clinician's impression of improvement. A stable outcome was defined as an unchanged MSG score or one that declined by <25% by the end of follow-up. An unresponsive outcome was defined as one of the following: (1) the MSG score increased (reflecting progressive infection); (2) progress notes reported relapsed or progressing infection; (3) patient stopped therapy because of intolerance or cost.

	0 months	3 months	6 months	12 months
Clinical signs	(1) Spinal pain (2) Paraparetic (3) Fever (4) Muscle wasting (5) Draining tract (6) Left flank fluctuant swelling (7) Lethargy	(1) Spinal pain (2) Paraparetic (3) Fever (4) Muscle wasting (5) Right flank fluctuant swelling	(1) Spinal pain (2) Right flank fluctuant swelling	(1) Spinal pain (2) Right flank fluctuant swelling

Radiographic findings	n/a	n/a	n/a	n/a

Serology titer	1 : 32	1 : 64	1 : 32	1 : 32

Modified MSG score	12	11—unresponsive	7—improved	7—stable

## References

[1] Greene C. E. (2011). *Infectious Diseases of the Dog and Cat*.

[2] Alanko K., Kahanpää A., Pätiälä J. (1975). The first two cases of coccidioidomycosis in Finland. *Journal of Internal Medicine*.

[3] Zalatnai A., Zala J., Sándor G. (1998). Coccidioidomycosis in Hungary. *Pathology & Oncology Research*.

[4] Fohlman J., Sjölin J., Bennich H., Chryssanthou E., Von Rosen M., Petrini B. (2000). Coccidioidomycosis as imported atypical pneumonia in Sweden. *Infectious Diseases*.

[5] Pistone T., Lacombe K., Poirot J. L., Girard P. M., Meynard J.-L. (2005). Imported concomitant coccidioidomycosis and histoplasmosis in an HIV-infected Colombian migrant in France. *Transactions of the Royal Society of Tropical Medicine and Hygiene*.

[6] Buitrago M. J., Cuenca-Estrella M. (2012). Current epidemiology and laboratory diagnosis of endemic mycoses in Spain. *Enfermedades Infecciosas Y Microbiologia Clinica*.

[7] Gobbi F., Angheben A., Farina C. (2012). Coccidioidomycosis: First imported case in Italy. *Journal of Travel Medicine*.

[8] College of Medicine Tucson Valley fever in dogs. http://www.vfce.arizona.edu/valley-fever-dogs/treatment.

[9] Catanzaro A., Friedman P. J., Schillaci R. (1983). Treatment of coccidioidomycosis with ketoconazole: An evaluation utilizing a new scoring system. *American Journal of Medicine*.

[10] Kim M. M., Vikram H. R., Kusne S., Seville M. T., Blair J. E. (2011). Treatment of refractory coccidioidomycosis with voriconazole or posaconazole. *Clinical Infectious Diseases*.

[11] Anstead G. M., Corcoran G., Lewis J., Berg D., Graybill J. R. (2005). Refractory coccidioidomycosis treated with posaconazole. *Clinical Infectious Diseases*.

[12] Pascual A., Calandra T., Bolay S., Buclin T., Bille J., Marchetti O. (2008). Voriconazole therapeutic drug monitoring in patients with invasive mycoses improves efficacy and safety outcomes. *Clinical Infectious Diseases*.

[13] Brüggemann R. J. M., Donnelly J. P., Aarnoutse R. E. (2008). Therapeutic drug monitoring of voriconazole. *Therapeutic Drug Monitoring*.

[14] Gold A. J., Langlois D. K., Refsal K. R. (2016). Evaluation of basal serum or plasma cortisol concentrations for the diagnosis of hypoadrenocorticism in dogs. *Journal of Veterinary Internal Medicine*.

[15] Willard M. D., Nachreiner R., McDonald R., Roudebush P. (1986). Ketoconazole-induced changes in selected canine hormone concentrations. *American Journal of Veterinary Research*.

[16] Marsden-Haug N., Goldoft M., Ralston C. (2013). Coccidioidomycosis acquired in Washington State. *Clinical Infectious Diseases*.

[17] Galgiani J. H., Ampel N. M., Blair J. E. (2005). Coccidioidomycosis. *Clinical Infectious Diseases*.

[18] Limper A. H., Knox K. S., Sarosi G. A. (2011). An official American Thoracic Society statement: treatment of fungal infections in adult pulmonary and critical care patients. *American Journal of Respiratory and Critical Care Medicine*.

[19] Somchit N., Norshahida A. R., Hasiah A. H., Zuraini A., Sulaiman M. R., Noordin M. M. (2004). Hepatotoxicity induced by antifungal drugs itraconazole and fluconazole in rats: a comparative in vivo study. *Human & Experimental Toxicology*.

[20] Somchit N., Ngee C. S., Yaakob A., Ahmad Z., Zakaria Z. A. (2009). Effects of cytochrome P450 inhibitors on itraconazole and fluconazole induced cytotoxicity in hepatocytes. *Journal of Toxicology*.

[21] College of Medicine Tucson Valley fever in dogs. http://www.vfce.arizona.edu/valley-fever-dogs/treatment.

[22] Prabhu R. M., Bonnell M., Currier B. L., Orenstein R. (2004). Successful treatment of disseminated nonmeningeal coccidioidomycosis with voriconazole. *Clinical Infectious Diseases*.

[23] Proia L. A., Tenorio A. R. (2004). Successful use of voriconazole for treatment of *Coccidioides* meningitis. *Antimicrobial Agents and Chemotherapy*.

[24] Cortez K. J., Walsh T. J., Bennett J. E. (2003). Successful treatment of coccidioidal meningitis with voriconazole. *Clinical Infectious Diseases*.

[25] Richardson K., Bell A. S., Dickinson R. P., Narayanaswami S., Ray S. J. UK-109,496, a novel, wide-spectrum triazole derivative for the treatment of fungal infections: synthesis and SAR.

[26] Sanati H., Belanger P., Fratti R., Ghannoum M. (1997). A new triazole, voriconazole (UK-109,496), blocks sterol biosynthesis in *Candida albicans* and *Candida krusei*. *Antimicrobial Agents and Chemotherapy*.

[27] Roffey S. J., Cole S., Comby P. (2003). The disposition of voriconazole in mouse, rat, rabbit, guinea pig, dog, and human. *Drug Metabolism and Disposition*.

[28] Atencia S., Papakonstantinou S., Leggett B., McAllister H., Mooney C. T. (2014). Systemic fungal infection in a dog: A unique case in Ireland. *Irish Veterinary Journal*.

[29] Bentley R. T., Faissler D., Sutherland-Smith J. (2011). Successful management of an intracranial phaeohyphomycotic fungal granuloma in a dog. *Journal of the American Veterinary Medical Association*.

[30] Taylor A. R., Young B. D., Levine G. J. (2015). Clinical features and magnetic resonance imaging findings in 7 dogs with central nervous system aspergillosis. *Journal of Veterinary Internal Medicine*.

[31] Ledbetter E. C., Starr J. K. (2015). Malassezia pachydermatis keratomycosis in a dog. *Medical Mycology Case Reports*.

[32] Newton E. J. W. (2012). Scedosporium apiospermum keratomycosis in a dog. *Veterinary Ophthalmology*.

[33] Nevile J. C., Hurn S. D., Turner A. G. (2015). Keratomycosis in five dogs. *Veterinary Ophthalmology*.

[34] Papich M. G. (2011). *Saunders Handbook of Veterinary Drugs*.

[35] FDA Approved Drug Products. https://www.accessdata.fda.gov/drugsatfda_docs/label/2010/021266s032lbl.pdf.

[36] den Hollander J. G., van Arkel C., Rijnders B. J., Lugtenburg P. J., de Marie S., Levin M.-D. (2006). Incidence of voriconazole hepatotoxicity during intravenous and oral treatment for invasive fungal infections. *Journal of Antimicrobial Chemotherapy*.

[37] FDA Approved Drug Products. http://www.fda.gov/ohrms/dockets/ac/01/briefing/3792b2_02_FDA-voriconazole.htm.

[38] Galgiani J. N., Ampel N. M., Blair J. E. (2016). 2016 Infectious Diseases Society of America (IDSA) clinical practice guideline for the treatment of coccidioidomycosis. *Clinical Infectious Diseases*.

[39] Ampel N. M. (2015). The treatment of coccidioidomycosis. *Revista do Instituto de Medicina Tropical de São Paulo*.

[40] Galgiani J. N., Catanzaro A., Cloud G. A. (2000). Comparison of oral fluconazole and itraconazole for progressive, nonmeningeal coccidioidomycosis: A randomized, double-blind trial. *Annals of Internal Medicine*.

